# Effect of mold enclosure and chisel design on fatigue bond strength of dental adhesive systems

**DOI:** 10.1111/eos.12864

**Published:** 2022-04-22

**Authors:** Wayne W. Barkmeier, Akimasa Tsujimoto, Mark A. Latta, Toshiki Takamizawa, Scott M. Radniecki, Franklin Garcia‐Godoy

**Affiliations:** ^1^ Department of General Dentistry Creighton University School of Dentistry Omaha Nebraska USA; ^2^ Department of Operative Dentistry University of Iowa College or Dentistry Iowa City Iowa USA; ^3^ Department of Operative Dentistry Nihon University School of Dentistry Tokyo Japan; ^4^ Department of Bioscience Research University of Tennessee Health Science Center Memphis Tennessee USA

**Keywords:** dental debonding, dental restoration failure, light‐curing of dental adhesives

## Abstract

To examine the effect of mold enclosure and chisel design on macro shear fatigue bond strengths of dental adhesive systems. The fatigue bond strength testing was conducted with two commercially available dental adhesive systems, (1) OptiBond eXTRa and (2) Scotchbond Universal, for bonding a resin composite (Filtek Supreme Ultra) to both enamel and dentin using a mold enclosure and a non‐mold enclosure with a knife‐edge and two sized notched‐edge chisel assemblies for loading. As a loading reference for the fatigue testing, macro shear bond strengths of the adhesive systems to enamel and dentin were conducted using a mold enclosure and a knife‐edge chisel assembly. The shear bond strengths with the mold enclosure using knife‐edge chisel assembly did not exhibit a significant difference between the adhesive systems for either enamel or dentin. The fatigue bond strengths of bonded specimens demonstrated significant differences when comparing the mold enclosure and non‐mold enclosure, but not between knife‐edge and notched‐edge chisel assemblies. The fatigue bond strengths of dental adhesive systems demonstrated significantly higher values when using mold‐enclosed bonded specimens than a non‐mold enclosure, regardless of type of chisel assembly.

## INTRODUCTION

As the interest in bonding of dental adhesive systems to enamel and dentin increased, so did the intensity across the globe of laboratory testing to measure bond strengths [1]. Most earlier studies focus on macro shear bond strengths [2]. This type of testing uses a monotonically increasing load to cylindrically shaped bonded specimens of more than 3 mm diameter until failure occurs [2]. The load was typically delivered using a knife‐edge chisel assembly to deliver the load to the bonded specimens. More recently, a notched‐edge assembly was developed by Ultradent to deliver the monotonically increasing load. This macro shear bond strength testing method using a notched‐edge assembly (Ultradent Notched‐Edge Testing Method) was adopted by the International Standards Organization in 2013.

While macro bond strength testing provides a good relative measure of adhesion characteristics, dynamic fatigue bond strength testing is generally considered a more relevant comparison to clinical situations [3]. Crack progression and failure in clinical situations are generally from repeated occlusal loading forces below the ultimate strength. In 2009, Erickson et al. [4] described the use of a mold enclosure of the bonded specimens for shear fatigue bond strength testing with a knife‐edge chisel assembly. In 2013 Cheetham et al. [5, 6] also reported on the advantages of a mold enclosure in macro and micro shear bond strength testing. The advantages reported for a mold enclosure included restriction of deformation as force is applied and the distribution of a point load source to minimize concentration of forces associated with a knife‐edge shearing assembly [5, 6].

In order to further evaluate the use of a mold enclosure with a notched‐edge assembly, the end piece on an Ultradent notched edge crosshead assembly was replaced with a machined piece that would accommodate a stainless‐steel mold enclosure ring. The purpose of this study was to examine if the mold enclosure and chisel design affected macro shear fatigue bond strength testing values. The first null hypothesis was that macro shear fatigue bond strengths using a mold enclosure and a non‐mold enclosure would not be different. The second null hypothesis was that macro shear fatigue bond strengths when using a knife‐edge or notched‐edge loading assembly would not be different.

## MATERIAL AND METHODS

The staircase method [7] was utilized to evaluate the effect of the chisel design and mold enclosure on macro shear fatigue bond strengths of dental adhesive systems to both enamel and dentin. The initial loading, using the staircase method, was approximately 50% of the measured macro static shear bond strength. The starting stress was determined by conducting preliminary macro shear bond strength testing.

This study, using non‐identified human molars, was reviewed by the Biomedical Institutional Review Board at Creighton University (No. 760765‐1) and it was determined that IRB approval was not required.

### Study materials

A two‐step self‐etch adhesive system, OptiBond eXTRa (2‐step SE, Kerr), and a universal adhesive, Scotchbond Universal (Universal Adhesive, 3 M Oral Care), were used in this laboratory study to bond a resin composite (Filtek Supreme Ultra, 3 M Oral Care) to both enamel and dentin. The adhesive systems (Table 1) were used for determining both macro static bond strengths and dynamic bond strengths.

**TABLE 1:** Materials used in the study

Code	Adhesive system	Main components	Manufacturer
Two‐step SE (Self‐etch)	OptiBond eXTRa Lot Numbers: Primer: 7247707 Adhesive: 7246204	Ethanol, 2‐hydroxyethyl methacrylate, glycerol dimethacrylate, glycerol phosphate dimethacrylate, sodium hexafluorosilicate	Dentsply Sirona
Universal adhesive	Scotchbond Universal Lot Number: 90712	2‐Hydroxyethyl methacrylate, bisphenol A diglycidyl ether dimethacrylate (BISGMA), 2‐propenoic acid, 2‐methyl‐, reaction products with 1, 10 decanediol and phosphoric oxide (P205), ethanol, water, 2‐propenoic acid, 2‐methyl‐, 3‐(trimethoxysilyl)propyl ester, reaction products with vitreous silica, copolymer of acrylic and itaconic acid, camphorquinone, dimethylaminobenzoate(‐4), (dimethylamino)ethyl methacrylate	3M Oral Care

### Shear bond strength testing

Macro shear bond strengths were measured using enamel and dentin surfaces that had been ground flat on non‐identified extracted human molars. The bonding surfaces were prepared by dividing the molars mesio‐distally before cutting off roughly two‐thirds of the root structure. The sectioned lingual and buccal molars were mounted in 25 mm phenolic rings with Triad DuaLine (Dentsply Sirona). The flat tooth surfaces were polished using a sequence of silicone carbide papers (Struers) under water coolant to a final surface with 4000‐grit paper.

Fifteen specimens were prepared for each group. The resin composite was bonded to the test surfaces using stainless‐steel metal rings with an inner diameter of 2.38 mm, an outer diameter of 4.70 mm and an edge thickness of 2.62 mm. The adherend side of the metal rings was treated with a releasing agent (3% solution of paraffin in hexane). The bonding sites on the ground flat molar surfaces were treated with the adhesive systems and the resin composite was then filled into the metal rings using a custom holding device. A Dentsply Spectrum 800 Curing Light (Dentsply Sirona) was used on the specimen surfaces with a curing time of 40 s. The specimens were stored for 24 h before testing, in distilled water at 37°C.

An Instron ElectroPuls E1000 (Instron) was utilized to load the specimens until failure with a crosshead speed of 1.0 mm/min. A metal rod knife‐edge chisel assembly was utilized to load the mold‐enclosed bonding specimens. Shear bond strengths (MPa) were measured from the peak load at failure divided by the bonded surface area. The fracture sites of the bonding specimens were examined following the debonding procedure to evaluate the type of bonding failure.

### Shear fatigue bond strength testing

**FIGURE 1:**
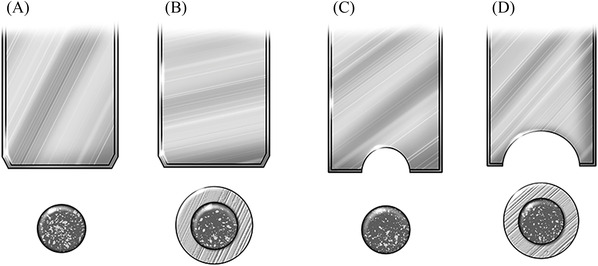
Four apparatus configurations (L to R) were used for fatigue testing: (A) knife‐edge chisel/non‐mold enclosure, (B) knife‐edge chisel/mold enclosure, (C) small notch chisel/non‐mold enclosure and (D) large notch chisel/mold enclosure

The specimens for macro shear fatigue bond strength testing were made in the same manner as described for the shear bond strength testing. For each of the two adhesive systems, twenty specimens were utilized for each of the following test conditions (Figure 1).

Knife‐edge chisel ‐ No mold enclosure

Knife‐edge chisel ‐ Mold enclosure

Notched‐edge chisel ‐ No mold enclosure

Notched‐edge chisel ‐ Mold enclosure

The bonded specimens for the knife‐edge and notched‐edge test conditions without a mold enclosure were prepared according to ISO 29022:2013 using the specified methodology. This resulted in a cylindrical bonded assembly 2.38 mm in diameter and 2.5 mm in height.

Stainless‐steel rings, as described for shear bond strength testing, were used for the two notched‐edge test conditions (2‐step SE and Universal Adhesive) with a mold enclosure. For the non‐mold‐enclosed specimens, the Ultradent notched‐edge crosshead assembly was used to deliver the loading stress. For the mold‐enclosed specimens, a special assembly was machined with the same design as the Ultradent fixture, but with a larger radius to accommodate the stainless‐steal rings (Figure 1).

The initial loading, using an Instron ElectroPuls E1000, for the staircase test method was set at approximately 50%–60% of the ultimate strength found in the shear bond strength testing (Table 2). The load was set with a lower limit of zero and a sine wave was used for 50,000 cycles at a frequency of 10 Hz or until failure occurred. After each test, the loading force was incrementally adjusted, increased for survival or decreased for failure, by approximately 10%. The loading force that produces 50% failures was calculated using a formula developed by Draughn [7] and is termed shear fatigue bond strength.

**TABLE 2:** Shear bond strengths and failure sites of dental adhesive systems to enamel and dentin using mold enclosure method with knife‐edge chisel assembly

Adhesive	Enamel Mean value (SD) MPa	Dentin Mean value (SD) MPa	Enamel failure sites	Dentin failure sites
Two‐step self‐etch adhesive	41.4 (8.9)^aA^	37.9 (10.8)^aA^	Adhesive‐80.0% Cohesive (Enamel)‐20.0%	Adhesive‐86.7% Mixed (Adhesive/Cohesive Dentin)‐13.3%
Universal adhesive	42.4 (5.4)^aA^	35.9 (11.4)^aA^	Adhesive‐73.3% Cohesive (Enamel)‐20.0% Mixed Adhesive/Cohesive Resin‐6.7%	Adhesive‐66.6% Mixed (Adhesive/Cohesive Resin)‐26.7 Cohesive Dentin‐6.6%

Different lower‐case letters within the same column indicate a significant difference (*p* < 0.05). Different capital case letters between columns indicate a significant difference (*p* < 0.05).

### Statistical analysis

A Shapiro‐Wilk test was utilized on the shear bond strengths to assess for conformance to a normal distribution followed by a two‐way ANOVA of the factors (1) adhesive (2‐step SE or Universal adhesive) and (2) substrate (enamel or dentin), followed by Tukey's post hoc test.

The statistical analysis of the fatigue bond strength data is not straightforward. As described above, 20 samples are used, of which approximately half fail, and the shear fatigue bond strength is calculated based on the stresses applied to the failed samples. Thus, there are no values from the samples that survived in the calculation. Nevertheless, the existence of the surviving samples is essential to the method. This means that, while the calculation gives a mean and standard deviation, it is not immediately clear whether the “sample size” for the purposes of analysis is the total number of samples (20), or the number of failed samples (which varies, but is normally close to 10). Dr. Martha E. Nunn, Department of Periodontics, Creighton University School of Dentistry, was consulted to devise an appropriate analysis method for this data, and this method was encoded in a custom spreadsheet, which was used to do the analysis for this experiment. It relies on a modified version of the *t*‐test, using the Bonferroni correction.

## RESULTS

### Shear bond strength

The results for the macro shear bond strengths to enamel and dentin can be found in Table 2. The Shapiro‐Wilk test confirmed data normality. The knife‐edge shearing assembly, with the mold‐enclosed bonding system, resulted in a shear bond strength to enamel of 41.4 MPa with 2‐step SE and 42.4 Mpa using Universal Adhesive (*p* > 0.05). The shear bond strength to dentin for 2‐step SE was 37.9 Mpa and 35.9 Mpa for Universal Adhesive (*p* > 0.05). These values were used to calculate the starting stress for fatigue bond strength testing.

The predominant failure types (Table 2) in both the enamel and dentin groups were adhesive failures, ranging from 66.6 to 86.7%. Cohesive failures in enamel were the same (20%) for both adhesive systems. For the dentin group 6.6% of the failures in Universal Adhesive were cohesive compared to none for the 2‐step SE.

### Shear fatigue bond strength

The results for macro shear fatigue bond strengths comparing knife‐edge and notched‐edge shearing assemblies, using both mold enclosure and non‐mold enclosure specimens for bonding to both enamel and dentin, can be found in Table 3.

**TABLE 3:** Shear fatigue bond strengths of dental adhesive systems to enamel and dentin

Adhesive	Chisel assembly	Mold enclosure	Enamel Mean value (SD) MPa	Dentin Mean value (SD) MPa
Two‐step self‐etch	Knife edge	No	9.5 (2.6)^aA^	11.3 (2.0)^aA^
	Knife edge	Yes	18.8 (3.0)^bA^	20.4 (3.7)^bA^
	Notched edge	No	9.7 (1.4)^aA^	11.8 (2.0)^aA^
	Notched edge	Yes	20.8 (3.3)^bA^	19.7 (3.6)^bA^
Universal adhesive	Knife edge	No	12.2 (1.2)^aA^	11.1 (1.3)^aA^
	Knife edge	Yes	20.6 (2.3)^bA^	17.5 (2.0)^bA^
	Notched edge	No	11.5 (1.7)^aA^	11.3 (1.7)^aA^
	Notched edge	Yes	16.3 (2.4)^bA^	16.5 (1.9)^bA^

Different lower‐case letters within columns indicate a significant difference (*p* < 0.05). Different capital case letters within rows indicate a significant difference (*p* < 0.05).

With the knife‐edge assembly, the enamel fatigue bond strengths for 2‐step SE were 9.5 MPa for non‐mold‐enclosed specimens and 18.8 MPa for mold‐enclosed specimens (*p* < 0.05). For Universal Adhesive, the fatigue bond strengths were 12.2 MPa for non‐mold enclosure and 20.6 MPa for mold enclosure (*p* < 0.05). With both adhesive systems, the mold enclosure values were significantly higher (*p* < 0.05) than the non‐mold enclosure strengths.

The dentin fatigue bond strengths for 2‐step SE with the knife‐edge and non‐mold enclosure were 11.3 MPa compared to 20.4 MPa for mold enclosure (*p* < 0.05). For Universal Adhesive and the knife‐edge assembly, the fatigue bond strength to dentin without mold enclosure was 11.1 MPa compared to 17.5 MPa (*p* < 0.05) with mold enclosure. For both adhesives, the fatigue bond strengths of the mold enclosure groups were significantly higher (*p* < 0.05) than the non‐mold enclosure groups.

With the notched‐edge assemblies, enamel fatigue bond strengths for non‐enclosed specimens using 2‐step SE were 9.7 MPa compared to 20.8 MPa for mold‐enclosed specimens (*p* < 0.05), compared to Universal Adhesive at 11.5 MPa for non‐enclosure and 16.3 MPa for mold enclosure (*p* < 0.05).

When the notched‐edge assembly fatigue bond strengths to dentin using 2‐step SE were compared, the non‐mold‐enclosed test condition was 11.8 MPa and the mold‐enclosed test condition was 19.7 MPa (*p* < 0.05). For Universal Adhesive, the fatigue bond strengths of the non‐mold enclosure specimens were 11.3 MPa and with mold enclosure the fatigue bond strength was 16.5 MPa (*p* < 0.05).

## DISCUSSION

Shear bond strength testing, using both macro (diameter of bonded area greater than 3 mm) and micro (diameter of bonded area less than 1 mm) sized specimens, has been utilized over the years to determine the bonding ability of dental adhesive systems to enamel and dentin substrates [2, 8]. Debonding techniques have included knife‐edge, notched‐edge, wire loop, and push‐out methods. More recently mold‐enclosed systems have been used in an effort to more evenly distribute the loading force. Barkmeier et al. [9] conducted macro shear bond strength testing to enamel using the Ultradent method and a mold‐enclosure method with a stainless‐steel ring. The diameter of the bonding sites was similar for both methods, but the shear bond strengths were approximately 20% higher for the mold enclosure method, when compared to the non‐mold enclosure Ultradent method. It was postulated that the force distribution with the mold enclosure yielded the higher bond strengths.

Laboratory fatigue testing of the adhesive bonds of dental restorative materials to enamel and dentin substrates is thought to yield more clinically relevant information than static bond strength testing [3]. While there is currently an International Standard (ISO 29022:2013E) for macro shear bond strength testing, technical guidelines for fatigue testing have not been specified at this time. In order to obtain additional information in the area of fatigue testing, the authors modified the current ISO shear bond strength methodology to include the mold enclosure.

The shear fatigue bond strengths (Table 3) of mold enclosure specimens typically generated significantly higher values (*p* < 0.05) for both enamel and dentin with both notched‐edge and knife‐edge chisel assemblies when compared to non‐mold enclosure specimens. The first null hypothesis, that macro shear fatigue bond strengths using a mold enclosure and a non‐mold enclosure would not be different, was rejected. An earlier finite element study [10] reported that shear bond strength testing with mold‐enclosed methods is more suitable than with non‐mold‐enclosed methods due to the elimination of heterogeneous stress. Cheetham et al. [5] reported that bond strengths for mold enclosure methods were significantly higher for a metal‐cement bond, regardless of the size of the adherend surface. Therefore, the results of this study are consistent with previous studies and clearly show the effectiveness of the mold enclosure method, even for tooth bonding and fatigue bond strength testing with macro sized specimens.

However, the shear fatigue bond strengths for enamel and dentin were not statistically different (*p* > 0.05) when the two types of chisel assemblies were compared (Table 3). Therefore, the second null hypothesis, that macro shear fatigue bond strengths when using a knife‐edge or notched‐edge loading assembly would not be different, was not rejected. An earlier study [11] using primary teeth showed that micro shear bond strength was not influenced by chisel design, and thus the present results are consistent with this previous work. It was assumed that the fatigue bond strength values differ depending on the contact area.

Lower fatigue bond strength was expected when the loading force was applied with point contact in the knife‐edge design due to the concentration of loading to the edge and non‐uniform stress with some tensile force. One of the most critical issues in fatigue bond strength testing is that the repeated loading force must be applied to the adhesive interface appropriately. In the present study, the chisel was positioned carefully to fit the stainless‐steel mold enclosure or the composite cylinder as closely as possible by a single operator, regardless of chisel design. This may be the reason why the chisel design did not seem to have any influence on fatigue bond strength values. Our fatigue bond strength data have been accumulated with a knife‐edge chisel over a period of more than 10 years and a large amount of data has already been published [12, 13]. The use of a notched‐edge chisel for fatigue bond strength testing has been suggested by some researchers, but the present study further validates the previous fatigue bond strength data gathered over the years with a knife‐edge chisel. A previously reported study [5] did show that a larger contact area with the adhesive interface reduced the stress concentration magnitude or mixed shear loading force to the adhesive interface. Thus, a notched‐edge chisel design assembly has been utilized in ISO shear bond strength testing. That is why a notched‐edge chisel assembly was expected to be useful for fatigue bond strength testing, but the data from the two types of chisel design were not significantly different.

Although not the focus of this study, it is interesting to note that no difference in the shear and fatigue bond strength was measured between the 2‐step self‐etch adhesive and the universal adhesive, regardless of substrate. This is consistent with the results of other studies [14, 15] showing that the fatigue bond strengths have given similar results using OptiBond XTR (Kerr) and Scotchbond Universal. It is also worth noting that clinical studies [16, 17] have not found any large differences in success rates between these two adhesives (OptiBond XTR for 6 years: 81.9%; Scotchbond Universal for 5 years: 81.4%), which may suggest that fatigue bond strength testing is clinically relevant. However, further evidence on that question is required.

The results of this study suggest that macro shear fatigue bond strength testing with the mold enclosure method, using either knife‐edge or notched‐edge loading assemblies, provides valuable evidence regarding the effectiveness of the bonding of adhesive systems with restorative materials and tooth structures. The results of this laboratory study provide valuable information regarding mold enclosure and chisel design for measuring the fatigue bond strength of adhesive systems and will allow better assessment of clinical options in the area of restorative dentistry. In addition, the ISO International Standard for adhesive testing might be revisited and possibly expanded in scope to include fatigue testing. This study clearly showed higher shear bond strength values with mold enclosure in both static and dynamic testing.

Based on overall results of this study, macro shear fatigue bond strength testing demonstrated that mold enclosure of an adhesively bonded resin composite to both enamel and dentin substrates resulted in higher fatigue bond strengths, when compared to non‐mold enclosure. In addition, when knife‐edge and notched‐edge shearing assemblies were compared in fatigue testing, there was no significant difference in fatigue bond strengths with mold‐enclosed specimens. These results suggest that the mold‐enclosed method for fatigue bond strength testing is preferable to non‐mold‐enclosed methods under these conditions. However, no difference was found between knife‐edge and notched‐edge shearing assemblies, which suggests that either could be used.

## CONFLICT OF INTEREST

The authors of this article certify that they have no propriety, financial, or other personal interests of any nature or kind in any product, service, and/or company that is presented in this article.

## AUTHOR CONTRIBUTIONS

**Conceptualization**: Akimasa Tsujimoto; **Methodology**: Wayne W. Barkmeier, Akimasa Tsujimoto; **Software**: Wayne W. Barkmeier, Akimasa Tsujimoto; **Validation**: Wayne W. Barkmeier, Akimasa Tsujimoto; **Formal Analysis**: Wayne W. Barkmeier; **Investigation**: Scott M. Radniecki; **Resources**: Toshiki Takamizawa, Mark A. Latta. **Data Curation**: Wayne W. Barkmeier, Akimasa Tsujimoto. **Writing—Original Draft**: Wayne W. Barkmeier, Akimasa Tsujimoto. **Writing—Review & Editing**: All authors. **Visualization**: Wayne W. Barkmeier, Akimasa Tsujimoto. **Project administration**: Mark A. Latta, Franklin Garcia‐Godoy.
